# Antiviral epithelial-macrophage crosstalk permits secondary bacterial infections

**DOI:** 10.1128/mbio.00863-23

**Published:** 2023-09-29

**Authors:** Sidney Lane, Tristan L. A. White, Erin E. Walsh, Richard T. Cattley, Rachel Cumberland, William F. Hawse, Greg M. Delgoffe, Stephen F. Badylak, Jennifer M. Bomberger

**Affiliations:** 1 Department of Microbiology and Molecular Genetics, University of Pittsburgh School of Medicine, Pittsburgh, Pennsylvania, USA; 2 Department of Immunology, University of Pittsburgh School of Medicine, Pittsburgh, Pennsylvania, USA; 3 Tumor Microenvironment Center, UPMC Hillman Cancer Center, Pittsburgh, Pennsylvania, USA; 4 McGowan Institute for Regenerative Medicine, University of Pittsburgh, Pittsburgh, Pennsylvania, USA; 5 Department of Surgery, University of Pittsburgh School of Medicine, Pittsburgh, Pennsylvania, USA; 6 Department of Bioengineering, University of Pittsburgh Swanson School of Engineering, Pittsburgh, Pennsylvania, USA; University of Washington, Seattle, Washington, USA

**Keywords:** extracellular vesicles, antiviral signaling, host-pathogen interactions, macrophage, respiratory epithelium

## Abstract

Extracellular vesicles (EVs) are produced by most known cell types as a form of intercellular communication to influence the physiological function of neighboring cells. During respiratory viral-bacterial coinfection, the preceding antiviral response can lead to an impaired antibacterial response, driven by miscommunication between cells responding to viruses and cells responding to bacteria. Previous studies have shown that antiviral signaling can influence EV cargo and promote antiviral defense in the recipient cell; however, how antiviral EVs may influence host defense against coinfecting microorganisms, specifically bacteria, is not known. Herein, we demonstrated that EVs released from the respiratory epithelium during antiviral signaling alter macrophage inflammatory signaling, induce anti-inflammatory metabolic reprogramming, and impair antibacterial activity against *Staphylococcus aureus*, a common coinfecting bacterial pathogen. Further proteomic analysis revealed that antiviral EVs are preferentially loaded with pyruvate kinase M2 (PKM2), a metabolic enzyme with immunomodulatory effects, and treatment with antiviral EVs leads to increased PKM2 in macrophages. Moreover, we showed that antiviral EV-treated macrophages displayed enhanced oxidative phosphorylation, a metabolic profile consistent with impaired *S. aureus* clearance, and that this metabolic state is phenocopied in macrophages treated with a PKM2 activator. Taken together, our findings identify EVs as a component of the epithelial antiviral response that contributes to impaired bacterial clearance through epithelial-macrophage crosstalk and suggest a role for EVs in driving disease progression during respiratory coinfection.

**IMPORTANCE:**

Miscommunication of antiviral and antibacterial immune signals drives worsened morbidity and mortality during respiratory viral-bacterial coinfections. Extracellular vesicles (EVs) are a form of intercellular communication with broad implications during infection, and here we show that epithelium-derived EVs released during the antiviral response impair the antibacterial activity of macrophages, an innate immune cell crucial for bacterial control in the airway. Macrophages exposed to antiviral EVs display reduced clearance of *Staphylococcus aureus* as well as altered inflammatory signaling and anti-inflammatory metabolic reprogramming, thus revealing EVs as a source of dysregulated epithelium-macrophage crosstalk during coinfection. As effective epithelium-macrophage communication is critical in mounting an appropriate immune response, this novel observation of epithelium-macrophage crosstalk shaping macrophage metabolism and antimicrobial function provides exciting new insight and improves our understanding of immune dysfunction during respiratory coinfections.

## INTRODUCTION

Respiratory infections are a leading cause of morbidity and mortality worldwide, and coinfections are associated with worsened disease outcomes (1, 2). In viral-bacterial coinfection, clinical and mechanistic studies show that a preceding acute respiratory viral infection promotes the establishment and exacerbation of subsequent bacterial infections, leading to increased morbidity (3, 4). Studies have revealed that much of the worsened pathogenesis observed during coinfection is the result of a dysregulated immune response driven by miscommunication between cells responding to either pathogen (3, 4). Indeed, it is well accepted that a preceding antiviral response can impair antibacterial activity in the respiratory tract. While advances have been made in understanding how the antiviral response limits antibacterial activity, the mechanisms mediating this dysfunction are not fully elucidated.

The respiratory epithelium is the primary replication target for most respiratory viruses and thus is responsible for initiating much of the antiviral response. Recognition of viral antigen via pattern recognition receptors leads to the induction of antiviral signaling, wherein the epithelium secretes numerous mediators to directly attack the virus or recruit immune cells to clear the site of infection. In the context of coinfection, however, these antiviral effectors can promote susceptibility to secondary bacterial infections (5). Epithelial antiviral signaling, driven primarily by type I and III interferon (IFN) production, contributes to increased bacterial burden via disruption of barrier integrity (6), decreased antimicrobial peptide secretion (7), and altered nutritional immunity (8). Critically, the epithelial antiviral response is also shown to alter the recruitment and activation of infiltrating immune cells, leading to reduced bacterial clearance (5). Given that much of the increased morbidity observed during coinfection is due to failed bacterial clearance, and that the epithelium is crucial for the recruitment and immunoeducation of cells in the airway, further research is needed to unravel the mechanisms by which the epithelial antiviral response contributes to the dysfunction of infiltrating immune cells and an impaired antibacterial response.

EVs are membrane-enclosed particles released from all known cell types as a form of intercellular communication. EVs are implicated in a wide range of cell processes where differential packaging of EV cargo from the origin cell, including proteins, nucleic acids, and lipids, influences activity in a recipient cell (9). EVs play a variable role during infection and, depending on the pathogen, can induce either pro- or anti-microbial programs through modulation of the recipient immune cell. Previous investigations of the antiviral response indicate that EVs released from cells primed by IFNs or the TLR3 agonist, polyinosinic-polycitidylic acid (poly(I:C)), are enriched with antiviral mediators and induce anti-viral defense in recipient cells (10 – 12). Recently, our group showed that acute viral infection of the epithelium, in part through induction of the antiviral response, alters EV cargo to promote bacterial growth (13), though it is not known how these EVs influence antibacterial immunity.

Reports suggest that the epithelium is a predominant producer of EVs in the airway, and that epithelium-derived EVs preferentially target macrophages (14, 15). Macrophages are the most abundant cell type in the airway lumen and a fundamental component of the innate immune response. During coinfection, macrophages are highly dysfunctional, and priming by the preceding antiviral response impairs antibacterial activity (16). Indeed, induction of IFN signaling and the subsequent downstream events are shown to suppress macrophage activation and bacterial clearance (17). As epithelium-macrophage crosstalk is critical for effective orchestration of the innate immune response, we sought to interrogate the role of epithelial EVs produced during the antiviral response in mediating macrophage antibacterial activity.

## RESULTS

### Poly(I:C) treatment of airway epithelial cells induces a robust antiviral response and does not influence the biophysical properties of extracellular vesicles

**FIG 1:**
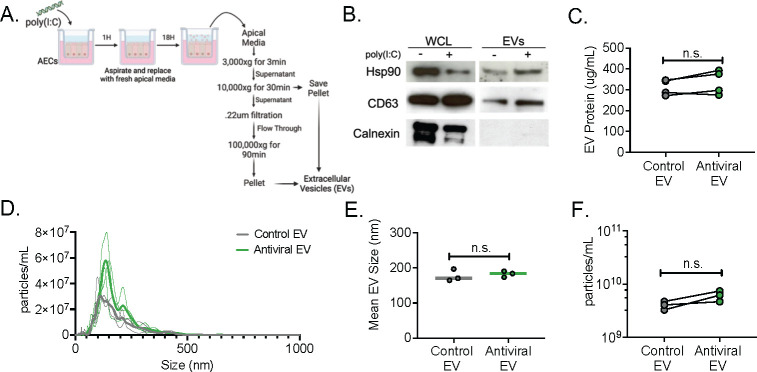
Characterization of antiviral AEC EVs. (**A**) Workflow of poly(I:C) stimulation and bulk EV isolation from well-differentiated AECs (16HBE14o- cell line). (**B**) Western blot analysis of positive (Hsp90, CD63) and negative (calnexin) EV markers and (**C**) quantification of total EV protein. NTA to evaluate EV (**D**) size distribution, (**E**) mean EV size, and (**F**) particle concentration. For all experiments, *n* ≥ 3. ns: not significant as evaluated by a paired *t*-test.

To determine whether EVs are a component of the epithelial antiviral response that contributes to an impaired antibacterial response, polarized airway epithelial cells (AECs) were stimulated with poly(I:C), a TLR3 agonist and viral mimetic, for 18 h to induce an antiviral response and collect secreted EVs into the apical lumen (Fig. 1A). TLR3 signaling is a crucial component of the immune response to many respiratory viruses identified during viral-bacterial coinfection (18, 19), and we confirmed that poly(I:C) treatment of AECs induced a vigorous antiviral response, driven by robust type I and type III IFN secretion (Fig. S1). EVs were isolated via differential filtration and centrifugation, with large (10,000 × *g* pellet) and small (100,000 × *g* pellet) EV populations combined to generate a bulk EV population, reminiscent of what would be present in the airways (Fig. 1A). EV purity was confirmed via protein analysis of positive (Hsp90, CD63) and negative (calnexin) EV markers, according to standardized protocols published by the International Society of Extracellular Vesicles (20) (Fig. 1B). No significant differences were observed in total protein concentration between control EVs (from untreated AECs) and EVs derived from poly(I:C)-treated AECs engaged in antiviral signaling (hereafter referred to as antiviral EVs) (Fig. 1C). The EV populations were shown to be free of large apoptotic bodies (particles ≥1,000 nm in diameter) (21) as assessed by size determination via nanoparticle tracking analysis (NTA) (Fig. 1D). Further characterization of EV mean particle size and particle concentration revealed no significant differences between the two EV populations (Fig. 1E and F). These data indicate that poly(I:C) stimulation of AECs has a limited effect on EV biophysical properties, suggesting that any observed phenotypes are likely driven by differences in EV cargo.

### Antiviral EV-stimulated macrophages display impaired clearance of *Staphylococcus aureus*

**FIG 2:**
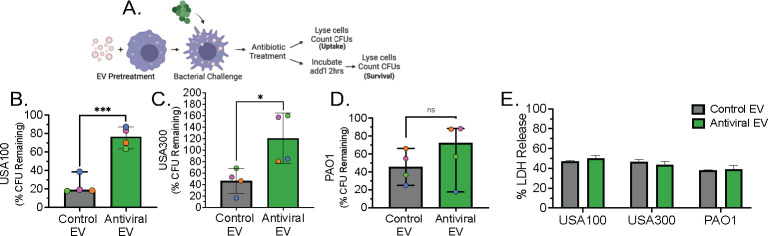
Exposure to antiviral AEC EVs impairs macrophage antibacterial activity. Human primary MDMs were pretreated with AEC EVs for 18 h and challenged with respiratory bacterial pathogens via an antibiotic protection assay. (**A**) Workflow of EV-MDM pretreatment and antibiotic protection assay. Antibiotic protection assay results displayed as Percent CFU Remaining {[(Avg. CFU Survival) / (Avg. CFU Uptake)] ∗ 100} for the common secondary respiratory bacterial pathogens (**B**) USA100, (**C**) USA300, and (**D**) PAO1. (**F**) Macrophage viability following bacterial challenge as measured by LDH release. Analyzed via paired *t*-test, data are presented as median ± range. ns: not significant; *P < 0.05; **P < 0.01; ***P < 0.001. For all experiments, *n* ≥ 4 paired samples, and each colored symbol denotes a donor.

To determine whether antiviral EVs (AEVs) alter the antibacterial response of macrophages, human monocyte-derived macrophages (MDMs) were treated with EVs and evaluated for bacterial clearance. During coinfection, macrophages present with a dysregulated antibacterial response marked by aberrant inflammatory signaling and impaired bacterial clearance (16). To determine whether EVs released during TLR3 engagement are a source of this dysregulation, we performed an antibiotic protection assay using the common respiratory secondary bacterial pathogens, *Staphylococcus aureus* and *Pseudomonas aeruginosa* (1, 2, 22). MDMs were isolated from healthy donor PBMCs and cultured for 8 d to generate resting (M0) macrophages. Macrophages were seeded in duplicate per condition and pretreated with EVs for 18 h. EV-stimulated macrophages were then challenged with bacteria for 30 min to allow for bacterial uptake before being treated with an antibiotic cocktail for 1 h to eliminate all extracellular bacteria. Afterward, one well was lysed and plated to count CFUs (Uptake), while the remaining well was incubated for an additional 2 h before being lysed and plated to count CFUs (Survival) (Fig. 2A). When evaluated as Percent CFU Remaining [(Avg. CFU Survival / Avg. CFU Uptake) ∗ 100], we find that *S. aureus* (USA100) survival is significantly increased in macrophages exposed to AEVs in comparison to those exposed to control EVs (CEVs) (Fig. 2B). Analysis of total CFU counts reveals that the impaired clearance is driven by significantly less bacterial uptake by AEV-treated macrophages (Fig. S2A). When evaluating the clearance of the hypervirulent community-acquired epidemic, *S. aureus* clone USA300, we observed a similar significant trend of impaired bacterial clearance in AEV-treated macrophages, driven by increased total CFU counts at the survival time point (Fig. 2C; Fig. S2B). This trend was observed when AEV-treated macrophages were challenged with *P. aeruginosa* (PAO1); however, the difference was not statistically significant (Fig. 2F, Fig. S2C). Assessment of macrophage viability revealed no differences for any condition, confirming that the observed phenotypes are not due to reduced macrophage survival (Fig. 2E). These data reveal that antiviral EVs diminish macrophage antibacterial activity, leading to impaired clearance of *S. aureus*.

### Macrophages exposed to antiviral EVs display robust interleukin 6 production

**FIG 3:**
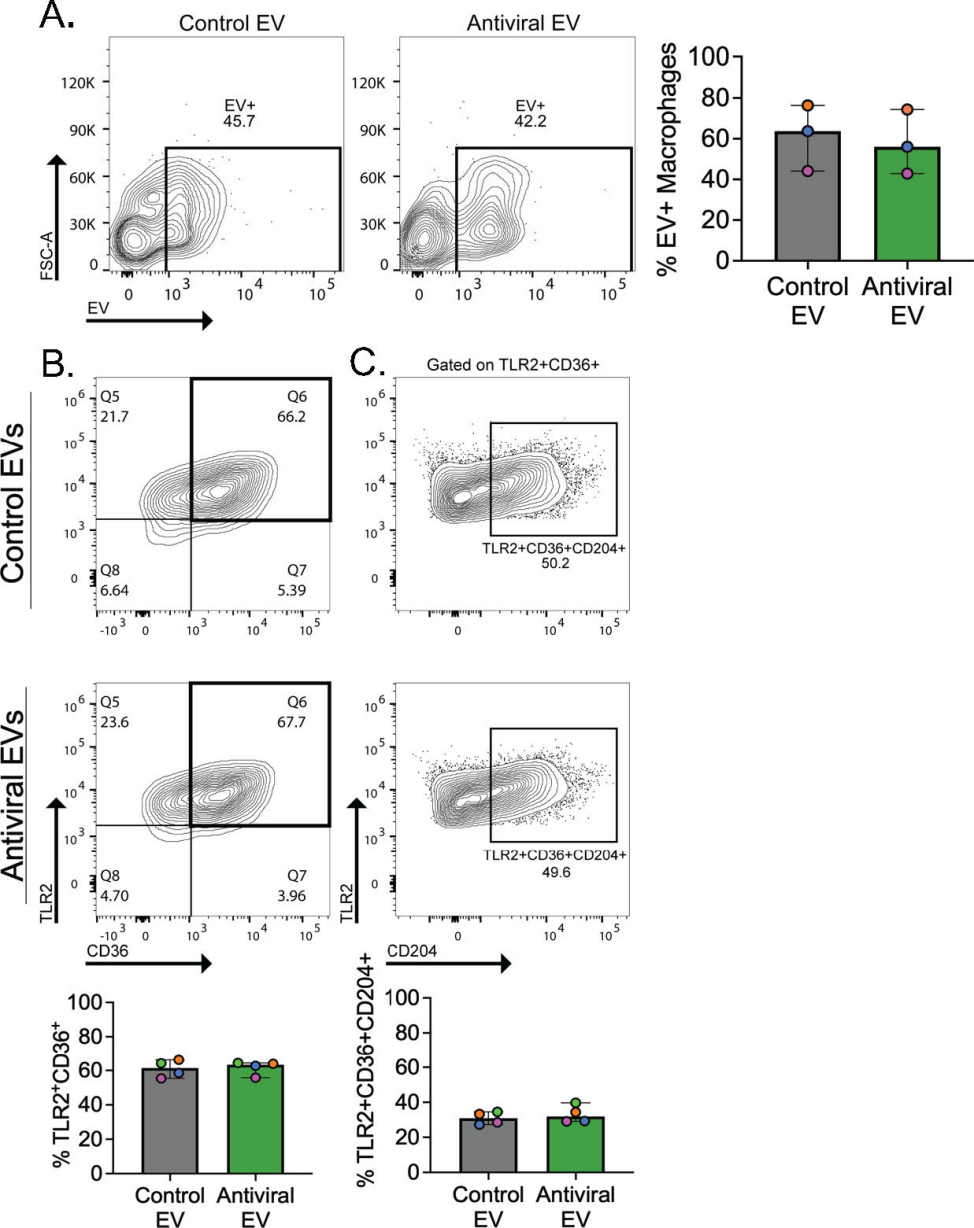
Exposure to antiviral AEC EVs does not alter macrophage surface marker expression. Flow cytometry analysis of (**A**) EV-macrophage association following 1 h of EV treatment is displayed as a representative contour plot with quantification to the right. Proinflammatory surface marker expression following 18 h of EV exposure for (**B**) TLR2^+^CD36^+^ cells and (**C**) TLR2^+^CD36^+^CD204^+^ cells, both displayed as representative contour plots with quantifications below. Each colored symbol denotes a donor. Data are presented as median ± range. For all experiments, *n* ≥ 3 paired samples.

As EV treatment altered macrophage antibacterial activity, we sought to characterize EV-macrophage interactions. We utilized flow cytometry to evaluate EV-macrophage association following 1 h of EV treatment and found no difference in the percentage of EV + macrophages between either EV population (Fig. 3A). We next evaluated macrophage polarization through the assessment of proinflammatory (TLR2, CD36, and CD204) and anti-inflammatory (Arg1, CD163, and CD206) macrophage markers after 18 h of EV treatment and *S. aureus* challenge. Macrophage polarization exists on a spectrum, ranging from a proinflammatory, highly microbicidal (M1) state to an anti-inflammatory, resolving (M2) state. While these classifications are effective in describing the general activation state of macrophages, *in vivo* studies have shown that the polarization state is highly plastic and influenced by environmental cues; thus, it is important to acknowledge that the M1 and M2 phenotypes likely represent extremes. As treatment with AEVs resulted in decreased bacterial uptake (Fig. S2A), we predicted decreases in TLR2, CD36, or CD204 in AEV-treated macrophages; however, there were no significant differences in single (Fig. S3E and F), double (Fig. 3B), or triple (Fig. 3C) positive expression of proinflammatory markers following treatment with either EV population. Similarly, no difference in anti-inflammatory markers was observed for any condition (Fig. S3). To determine whether differences in expression may be mediated via *S. aureus*, we also evaluated surface marker expression following EV treatment and *S. aureus* challenge, though no significant differences were observed (Fig. S4).

**FIG 4:**
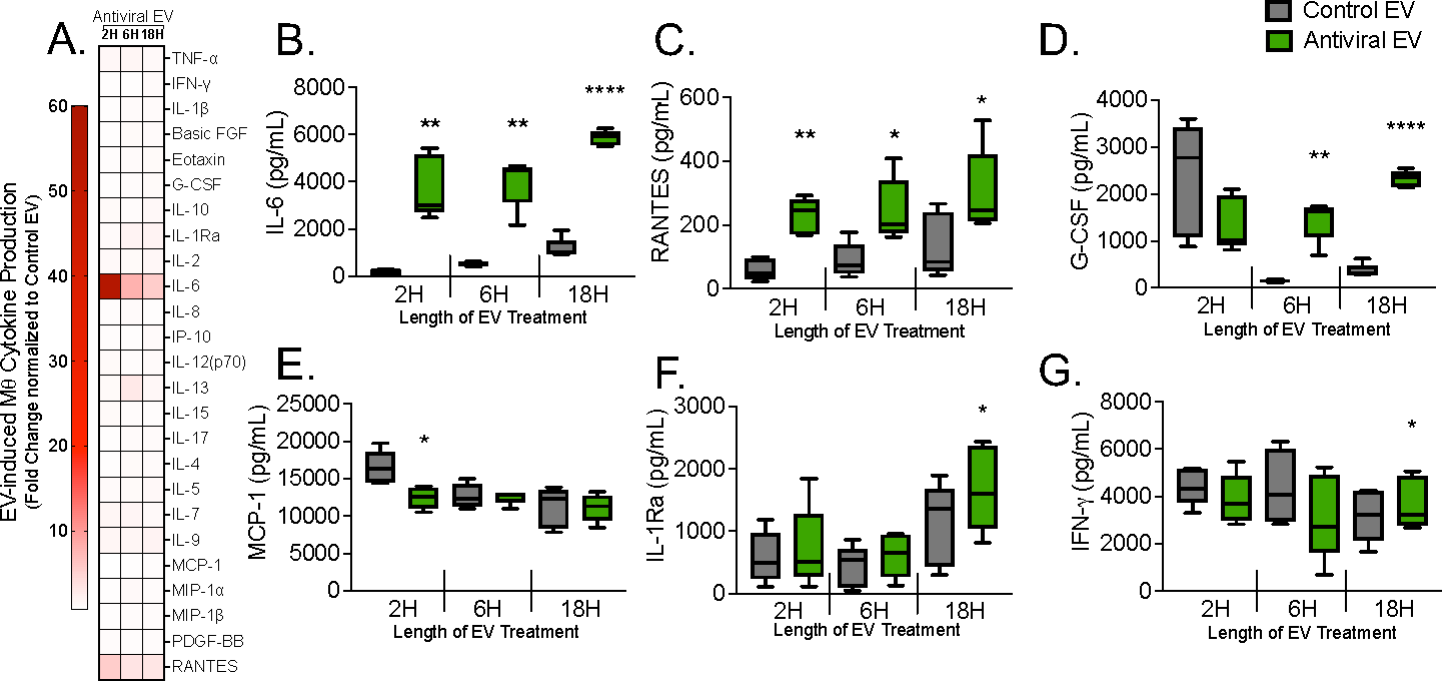
Exposure to antiviral AEC EVs induces inflammatory cytokine production in macrophages. Multiplexed cytokine array of macrophage cytokine production following EV pretreatment. Macrophage supernatant was collected 2, 6, and 18 h post-treatment with control or antiviral EVs and analyzed via multiplex analysis. (**A**) Heat map of all probed cytokines, displayed as fold change normalized to CEV-treated macrophages. (**B–G**) Significantly different cytokines as determined by concentration values. Gray bars: control EVs; green bars: antiviral EVs. Data are displayed as a box plot with the black horizontal line representing the median and whiskers extending to the minimum and maximum values. Analyzed via paired *t*-testns: not significant; *P < 0.05; **P < 0.01; ***P < 0.001; ****P < 0.0001. For all experiments, *n* ≥ 4 paired donors.

Macrophage polarization can also be evaluated via cytokine production. Macrophages were treated with EVs for 2, 6, or 18 h, and the supernatants were collected for multiplex analysis. Of the 27 cytokines probed, only interleukin 6 (IL-6) was found to have a fold change increase ≥5 for all time points when normalizing AEV-treated macrophages to controls (Fig. 4A). Analysis of cytokine concentration revealed five additional proinflammatory cytokines that were significantly increased in AEV-treated macrophages at any time point, though RANTES was the only cytokine in addition to IL-6 to display significant differences at all time points (Fig. 4). Surprisingly, a significant difference in IL-6 production was not observed following *S. aureus* challenge (Fig. S4), suggesting that AEV-mediated IL-6 production does not directly mediate the decrease in bactericidal activity. Notably, IL-6 is one of the most highly induced cytokines during viral-bacterial coinfection and is associated with worsened disease outcome (23 – 25). Importantly, production of cytokines canonically induced by direct poly(I:C) treatment of macrophages, namely, tumor necrosis factor alpha (TNFα) (26, 27) and interferon-γ induced protein 10 (IP-10) (27, 28), was not upregulated (Fig. 3A), giving additional confidence that the observed phenotype is not due to contaminating poly(I:C) molecules in the AEV population. Taken together, these data show that AEVs induce proinflammatory cytokine signaling in macrophages, though they do not induce overt changes in polarization state.

### Antiviral EVs are enriched with metabolic enzymes, including pyruvate kinase M2

**FIG 5:**
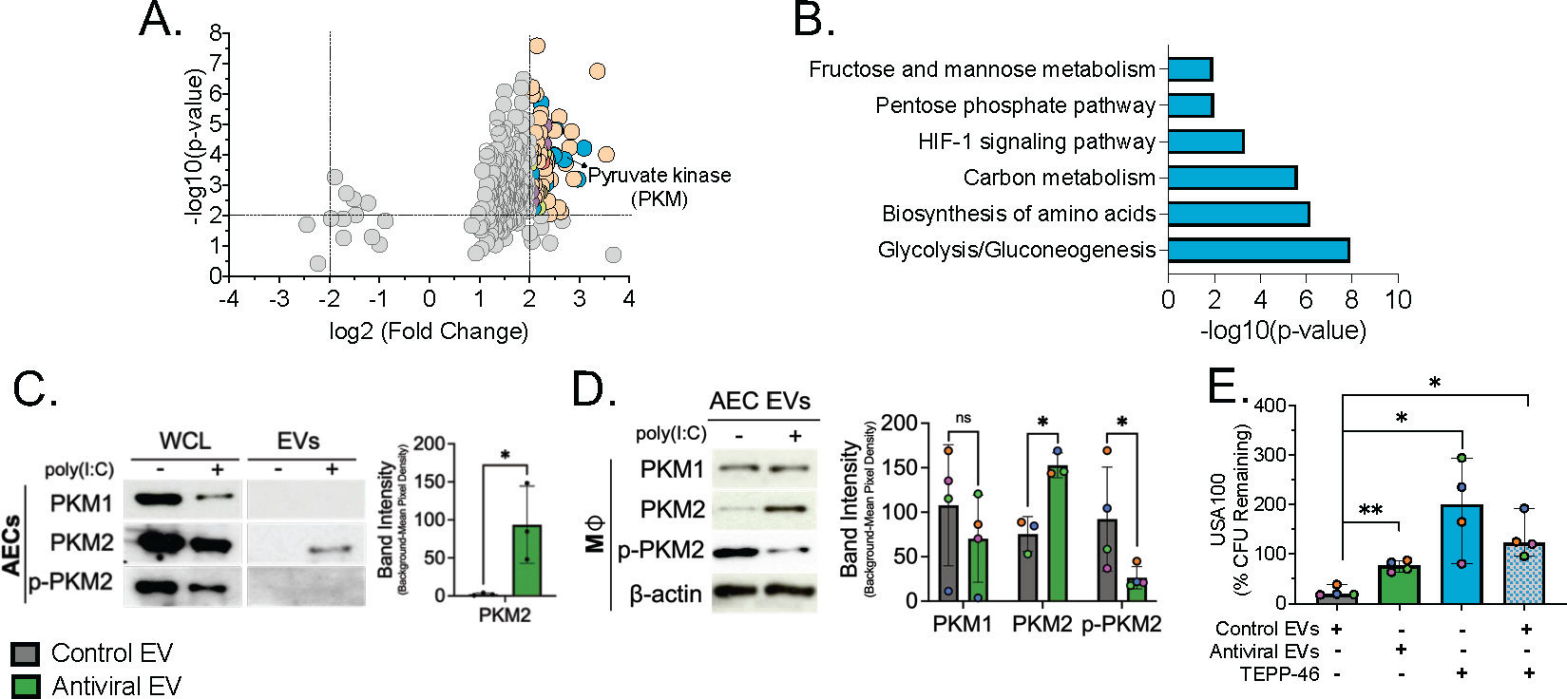
PKM2 is enriched on antiviral AEC EVs. Label-free quantitative mass spectrometry of AEC EVs reveals differences in protein cargo loading following poly(I:C) treatment of AECs. (**A**) Volcano plot of all proteins identified with high confidence in CEVs and AEVs. Significantly differentially abundant proteins in antiviral EVs (≥2.0 threshold cut-off), with colored symbols indicating proteome category as determined by KEGG pathway analysis (tan, genetic information processing; blue, metabolism; purple, cellular processing; light green, environmental information processing; red, organismal systems). (**B**) Gene Ontology categories following pathway enrichment of metabolic proteins in AEVs as determined by DAVID Bioinformatic Database. Representative western blot of PKM isoforms on (**C**) AEC EVs and (**D**) macrophages following 18 h of EV pretreatment. Band intensities are quantified to the right. To determine if PKM2 activation is sufficient to impair macrophage antibacterial activity against *S. aureus*, macrophages were pretreated with TEPP-46, a PKM2 activator, and challenged with *S. aureus* in an antibiotic protection assay. (**E**) Results from the antibiotic protection assay are displayed as % CFU Remaining. Comparisons between two groups were analyzed via paired *t*-test. Comparisons between multiple groups were analyzed via RM one-way analysis of variance with Geisser-Greenhouse correction. Data are presented as median ± range. ns: not significant; *P < 0.05; **P < 0.01; ***P < 0.001. For all experiments, *n* ≥ 3 paired samples. Each colored symbol denotes a donor.

As the characterization of macrophage polarization did not reveal a cause of the impaired antibacterial activity, we next evaluated EV cargo composition to probe the mechanism of reduced *S. aureus* clearance. We utilized label-free quantitative mass spectrometry to evaluate differences in common proteins found in AEVs versus CEVs. We detected over 2,500 proteins, with 326 proteins identified with high confidence. Of those, 69 proteins were found to have significantly increased differential abundance in AEVs versus CEVs, with no proteins found to be significantly decreased (Fig. 5A). Kyoto Encyclopedia for Genes and Genomes (KEGG) pathway analysis binned these proteins into five different proteome categories, with the majority of proteins involved in genetic information processing or, most interestingly, metabolism (Fig. 5A, tan or blue symbols, respectively). Both viral infection and antiviral signaling are shown to mediate shifts in glycolytic programming, and EVs have been previously reported to carry metabolic enzymes to influence activity in the recipient cell (29 – 33). Furthermore, metabolic state is a critical regulator of macrophage polarization and antibacterial activity (34). Considering this, we sought to further characterize the metabolic enzymes enriched on AEVs for a role in modulating macrophage antibacterial activity.

Pathway analysis of the metabolic proteins revealed enrichment of glycolytic pathways (Fig. 5B), with pyruvate kinase M (PKM) being the most highly enriched glycolytic enzyme found on AEVs (Fig. 5A). Pyruvate kinase acts in the final rate-limiting step of glycolysis, regulating the conversion of phosphoenolpyruvate to pyruvate to produce ATP, and has been extensively studied for its role in immunometabolism (35, 36). Several isoforms exist, including PKM1 and PKM2, wherein PKM1 exists as a stable enzymatically active tetramer, while PKM2 shifts between an enzymatically inactive dimer and an enzymatically active tetramer (35, 36). Inactive PKM2 has been extensively studied for its role in promoting aerobic glycolysis in cancer cells as well as for its non-canonical roles as a protein transactivator and protein kinase (35, 36). When performing its non-canonical functions and upon phosphorylation, inactive PKM2 can translocate to the nucleus and induce proinflammatory gene expression (35, 37). Accordingly, enforcement of active PKM2 through the use of small-molecule activators is shown to abrogate proinflammatory signaling. To determine which PKM isoforms are loaded onto AEVs, we analyzed EV protein via western blot for PKM1, PKM2, and p-PKM2(Tyr105) as an evaluation of the inactive nuclear fraction, as has been previously described (37, 38). Neither PKM1 nor p-PKM2(Tyr105) were detected in either EV population, indicating that PKM2 is exclusively loaded onto AEVs, primarily in its active form (Fig. 5C). When we evaluated macrophage PKM expression after EV treatment, we found that macrophages treated with AEVs had significantly increased PKM2 expression and a concurrent, proportionately significant decrease in p-PKM2(Tyr105) expression in comparison to macrophages exposed to CEVs (Fig. 4D). No differences were detected in PKM1 expression (Fig. 5D). Thus, exposure to AEVs is associated with a shift toward active PKM2 in macrophages.

### Activation of PKM2 in macrophages leads to impaired clearance of *S. aureus*

In macrophages, inactive PKM2 is shown to be crucial for proinflammatory signaling in response to lipopolysaccharide (LPS) stimulation (39, 40). Activation of PKM2 using small molecule activators was shown to dampen inflammatory signaling and decrease clearance of *Mycobacterium tuberculosis* and *Salmonella typhimurium* (39). To determine whether activation of PKM2 is sufficient for impaired clearance of *S. aureus*, we pretreated macrophages with TEPP-46, a PKM2 activator (39, 41), and performed an antibiotic protection assay. Indeed, TEPP-46 induced specific activation of PKM2 in macrophages (Fig. S5B) and led to a significant increase in *S. aureus* survival in comparison to those treated with CEVs (Fig. 5E). As macrophages exposed to CEVs display effective control of *S. aureus* (Fig. 2B) and predominantly express inactive PKM2 (Fig. 5D), we wanted to evaluate whether promotion of active PKM2 via TEPP-46 treatment would abrogate this phenotype. Strikingly, activation of PKM2 is sufficient to overcome the antibacterial effect of CEV exposure, as macrophages treated with TEPP-46 + CEVs have significantly increased *S. aureus* survival in comparison to those treated with CEVs only (Fig. 5E). These data reveal that PKM2 activation is sufficient to impair macrophage activity against *S. aureus*.

### Exposure to antiviral EVs induces an anti-inflammatory metabolic profile in macrophages

**FIG 6:**
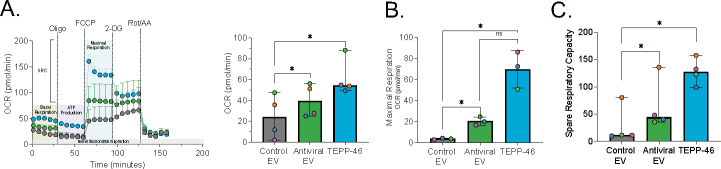
Antiviral EVs promote an anti-inflammatory metabolic state in macrophages. Metabolic flux analysis of macrophages after pretreatment with EVs or TEPP-46. (**A**) Kinetic graph and quantification of macrophage OCR with quantification of (**B**) maximal respiration and (**C**) spare respiratory capacity. Each colored symbol denotes a donor. Analyzed by RM one-way analysis of variance with Geisser-Greenhouse correction, data are presented as median ± range. ns: not significant; *P < 0.05; **P < 0.01; ***P < 0.001. For all experiments, *n* ≥ 3 paired samples.

Inactive PKM2 has been well described for its role in promoting aerobic glycolysis, while active PKM2 is typically associated with the enforcement of mitochondrial oxidative phosphorylation (OXPHOS) (35, 36, 39, 40). In macrophages, the switch to aerobic glycolysis is a hallmark of M1 polarization and increased antibacterial activity, while enhancement of OXPHOS drives reduced microbicidal activity and resolves inflammation (42). Indeed, enhancement of OXPHOS in macrophages is a key feature of chronic *S. aureus* infection, contributing to host evasion and bacterial persistence (43 – 45). Thus, we hypothesized that AEVs may be promoting an anti-bactericidal metabolic program leading to impaired *S. aureus* clearance. To evaluate the metabolic profile of macrophages after EV treatment, we performed a mitochondrial stress test via the Seahorse XFe96 Bioanalyzer. We analyzed OXPHOS through the assessment of oxygen consumption rate (OCR) and found that macrophages pretreated with AEVs had significantly increased OCR in comparison to those treated with CEVs (Fig. 6A). Further evaluation revealed that the difference in OCR was driven by significantly increased maximal respiration rates and spare respiratory capacity (Fig. 6B and C). Importantly, TEPP-46 treatment induced a similar metabolic profile as AEVs (Fig. 6A through C), suggesting that in AEV-treated macrophages, the shift toward tetrameric/active PKM2 may be driving an anti-inflammatory metabolic profile. No differences in glycolysis, as evaluated by extracellular acidification rate, were observed for any condition (Fig. S6). Taken together, these data are consistent with the conclusion that AEVs induce an anti-inflammatory metabolic profile in macrophages, which leads to dysfunctional antibacterial killing during coinfection.

## DISCUSSION

EVs are a form of intercellular communication that has been unexplored in the context of coinfection, where miscommunication of antiviral and antibacterial signals is a significant source of dysregulation during respiratory viral-bacterial coinfection. Given the significant morbidity and mortality associated with respiratory coinfections, furthering our understanding of the immunological mechanisms that contribute to disease progression is prudent.

The rationale for this investigation stemmed from our previous study that reported EVs released from virus-infected and poly(I:C)-stimulated epithelial cells promote bacterial growth during coinfection (13). Given that EVs have been extensively studied for their immunomodulatory effects (9), this investigation aimed to define how EVs released as part of the antiviral response influence the antibacterial activity of macrophages, one of the predominant innate immune cells in the respiratory tract. Our findings demonstrate that epithelial EVs released as part of the antiviral response are key factors in shaping the antibacterial response and metabolic state of the innate immune system during coinfection.

Macrophage dysfunction during viral-bacterial coinfection is driven by aberrant inflammatory signaling and impaired bacterial clearance, and herein, we provide evidence that antiviral EVs contribute to this dysfunction. We found that AEVs induced macrophage production of the pleiotropic proinflammatory cytokine IL-6. IL-6 is essential for effective control of acute respiratory viruses (46) and is one of the most significantly differentially expressed cytokines in co- vs. mono-infection (23, 24, 47, 48). During coinfection, IL-6 is reported to have both a protective (48) and detrimental role, though excessive IL-6 production is associated with increased inflammation, host cell damage, and bacterial burden and is considered a biomarker of worsened disease outcome (49). Macrophages and monocyte-derived cells are major producers of IL-6 in the airway; thus, our findings suggest that AEVs could contribute to aberrant inflammatory signaling during coinfection through the induction of macrophage-derived IL-6.

Metabolic regulation of macrophage activation has been well described (34). Exposure to AEVs led to enhanced OXPHOS in macrophages, a metabolic signature associated with limited microbicidal activity and inflammation resolution. Importantly, several recent studies investigating epithelial and macrophage metabolism during antiviral signaling indicate that enforcement of OXPHOS can promote antiviral cytokine production, including IL-6, suggesting that under certain conditions, OXPHOS may promote inflammation (27, 50). When challenged with *S. aureus*, we observed that AEV-treated macrophages were unable to control infection in comparison to their counterparts exposed to CEVs, in keeping with the enforcement of OXPHOS leading to reduced antibacterial activity. Importantly, *S. aureus* can act directly on macrophages to modify polarization and enforce OXPHOS in macrophages, a hallmark of chronic *S. aureus* infection, driving immune evasion and bacterial persistence (43 – 45). When considering these findings, it is tempting to speculate that while AEV enforcement of OXPHOS may be beneficial for the antiviral response, whether limiting or promoting inflammatory signaling, in the event of a secondary bacterial infection, AEV-primed macrophages are at a unique disadvantage to defend against *S. aureus*.

Previous studies investigating the antiviral response have shown that EVs released from cells primed by poly(I:C) or IFNs are enriched with antiviral mediators (10 – 12). In our study, the metabolic enzyme PKM2 was exclusively loaded onto AEVs. Metabolic enzymes, including PKM2, have been previously reported to be loaded on EVs and influence the metabolic state of recipient cells (30 – 32). Our study identified TLR3 stimulation as a novel mechanism by which active PKM2 is loaded onto epithelial EVs. Why TLR3 stimulation would result in the packaging of active PKM2 into EVs remains to be determined. PKM2 is a major regulator of immunometabolism, with the inactive and active forms being associated with proinflammatory or anti-inflammatory metabolic programs, respectively. Studies have indicated that antiviral signaling requires aerobic glycolysis (33), a metabolic state that would favor inactive PKM2. It is possible that loading active PKM2 onto EVs during antiviral signaling is an epithelial mechanism to rid the cell of the anti-inflammatory active PKM2 and facilitate the switch to inactive PKM2 and the subsequent proinflammatory response. Alternatively, loading active PKM2 onto EVs could be a mechanism to moderate inflammation in recipient cells. Macrophages exposed to AEVs expressed significantly more active PKM2 and significantly decreased inactive PKM2 in comparison to macrophages exposed to CEVs. Previous macrophage studies show that a shift toward active PKM2 abrogates inflammatory signaling (39, 40), suggesting that the AEV-mediated shift to active PKM2 could be a mechanism to prevent excess inflammation during the antiviral response. Questions remain regarding the molecular mechanisms of EV-derived PKM2. Primarily, how is PKM2 loaded onto epithelial EVs during antiviral signaling? Additionally, by what mechanism do AEVs drive a shift in PKM2 expression in macrophages? Through direct delivery of EV-derived PKM2 or, alternatively, an AEV-mediated signaling event? Addressing these questions will require additional studies that are outside the scope of the current investigation.

Activation of PKM2 in macrophages has previously been shown to impair clearance of bacterial pathogens (39), and we are building on this literature by reporting that activation of PKM2 worsens clearance of *S. aureus*. We demonstrated that activation of PKM2 with the small molecule activator, TEPP-46, is sufficient to impair clearance of *S. aureus*. Furthermore, we observed that macrophages treated with AEVs or TEPP-46 displayed similar metabolic profiles, both inducing an enforcement of OXPHOS. Activation of PKM2 is shown to enforce OXPHOS, suggesting that the AEV-derived PKM2 is driving macrophage metabolic reprogramming and the subsequent impairment of *S. aureus* clearance. Critically, it is important to note that PKM2 was not the only differentially expressed protein on AEVs. Our data indicate that 68 other proteins are also differentially abundant when comparing AEVs to CEVs, notwithstanding the nucleic acids, lipids, metabolites, and other cargo that are differentially loaded onto AEVs. It is likely that while PKM2 may be driving the observed macrophage phenotypes, it is working in concert with the other cargo loaded onto AEVs to influence macrophage activity.

Our study is not without limitations. The use of primary human MDMs strengthened the clinical translatability of our study; however, it also restricted the sample size and power of the study. A larger sample size could reveal differences that our current study was underpowered to detect. Additionally, while the use of a viral mimetic allowed for the interrogation of the specific contributions of the antiviral EV response to macrophage dysfunction, during active viral infection, the presence of a live virus will add an additional layer of complexity. Epithelial EVs released from cells infected with influenza or human rhinovirus, common coinfecting respiratory viruses, are reported to induce either pro- or anti-viral effects in recipient cells (51). Furthermore, specific strains of both viruses have been reported to establish productive infection in macrophages (52, 53). Thus, when we consider how our findings may translate *in vivo*, it is clear that AEVs are one component of a cascade of signals encountered by infiltrating macrophages during respiratory coinfection that regulate macrophage function.

Effective cellular communication is crucial for mounting an appropriate immune response. This is emphasized during viral-bacterial coinfection, wherein worsened disease outcomes are driven by miscommunication between cells responding to either pathogen. Here, we provided evidence that EVs released during antiviral signaling in respiratory epithelial cells influence macrophage activity and reduce clearance of *S. aureus*, a common secondary respiratory bacterial pathogen. These findings identify EVs produced during antiviral signaling as a source of impaired antibacterial activity. When taken with our previous findings that indicated AEC EVs released during coinfection promote bacterial growth (13), our studies provide robust evidence that epithelial EVs are a significant source of dysregulation during coinfection and warrant further investigation into the role of EVs during these devastating infections.

## MATERIALS AND METHODS

### Cell culture

Immortalized 16HBE14o- cells (human bronchial epithelial cell line; ATCC) were cultured in minimal essential media (MEM) supplemented with 10% fetal bovine serum (FBS), 2 mM L-glutamine, 5 U/mL penicillin–5 μg/mL streptomycin (P/S), and 0.5 mg/mL Plasmocin prophylactic at 37°C in 5% CO_2_. Cells were seeded on 24 mm Transwell filters for 12–14 d at the liquid-liquid interface. On days 12–14, cells were treated apically with 100 µg/mL high molecular weight poly(I:C) (InvivoGen) in MEM for 1 h. Afterward, the apical media were replaced with 1 mL fresh MEM, and the cells were incubated for an additional 18 h. During poly(I:C) treatment, cells were cultured in MEM supplemented with 10% exosome-free FBS (System Biosciences) and 2 mM L-glutamine. Human MDMs were generated from deidentified donor peripheral blood mononuclear cells (PBMCs) purchased from Vitalant. Briefly, PBMCs were isolated from blood via a Ficoll-density gradient, and monocytes were negatively selected from PBMCs using the Classical Monocyte Isolation Kit (Miltenyi) via Magnetic-Activated Cell Sorting (Miltenyi). Isolated monocytes were cultured with 100 ng/mL recombinant human macrophage colony-stimulating factor (rhM-CSF; R&D) in RPMI-1640 media supplemented with 10% heat-inactivated FBS, 2 mM GlutaMAX-I, and 5 U/mL–5 µg/mL P/S for 8 d to generate resting (M0) macrophages. On day 7, macrophages were treated with 50 µL EVs for 18 h, 50 µM TEPP-46 (PKM2 activator; Millipore) for 24 h, or both treatments simultaneously, where indicated. Twenty-four hours prior to all treatments (day 6 of macrophage culture), macrophages were washed with phosphate buffered saline (PBS) and cultured in RPMI-1640 media supplemented with 100 ng/mL rhM-CSF, 10% exosome-free FBS, and 2 mM GlutaMAX-I.

### Extracellular vesicle isolation

16HBE14o- apical EVs were collected following poly(I:C) stimulation and isolated through differential filtration and centrifugation according to an established protocol (20). Briefly, apical secretions were successively centrifuged at 1,400 × *g* for 3 min and 10,000 × *g* for 30 min. The supernatant was filtered through a 0.22-µm filter (Millipore) and then centrifuged at 100,000 × *g* for 90 min using a SW-60 rotor (Beckman). The 100,000 × *g* and 10,000 × *g* pellets were then combined in 1 mL PBS (6× concentration compared to apical secretions), resulting in a bulk EV population. All centrifugation steps were done at 4°C. EV size distribution and concentration were quantified by Nanosight NS300 (Malvern Panalytical) using a green 532 nm laser. Briefly, EVs were diluted 1,000-fold in 0.1 µm of filtered diH_2_O. The diluted particles were continuously injected by a syringe pump into the Nanosight NS300 view field. Particles were individually recorded and tracked for three 45-s captures for each sample. All frames captured were analyzed by NTA version 3.4 analysis software.

### Protein quantification and western blot

Protein concentrations of epithelial cell and macrophage whole cell lysates were quantified using the Pierce 660 nm Protein Assay Kit (Thermo), and protein concentrations of EVs were quantified using the Micro BCA Protein Assay Kit (Thermo), both according to the manufacturer’s protocol. Proteins were separated by SDS-PAGE on Tris gels (Bio-Rad) and transferred onto poly(vinylidene fluoride) (PVDF) membranes (Bio-Rad). The following antibodies were used for protein detection: anti-Hsp90 (Enzo Life Sciences), anti-CD63 (Santa Cruz Biotechnology), anti-calnexin (Santa Cruz Biotechnology), anti-PKM1 (Cell Signaling Technology), anti-phospho-PKM2(Tyr105) (Cell Signaling Technology), anti-PKM2 (Cell Signaling Technology), and anti-β-actin (Santa Cruz Biotechnology). Secondary antibodies were goat anti-mouse, goat anti-rabbit, and rat anti-goat conjugated to horseradish peroxidase (Bio-Rad).

### Bacterial strains

Hospital-associated methicillin-resistant *Staphylococcus aureus* (MRSA) USA100 Tokyo clone and community-associated epidemic MRSA clone USA300 LAC were each cultured overnight in tryptic soy broth at 37°C with shaking. PAO1 was grown overnight in Lysogeny Broth at 37°C with shaking. All strains were normalized to OD = 0.05 in PBS^−/−^ prior to macrophage assays.

### Antibiotic protection assay

Macrophages were cultured for 8 d, as described above, in duplicate per condition in 48-well non-tissue culture-treated plates. Macrophages were challenged with respective bacteria (MOI = 10–20) and centrifuged at 500 × *g* for 5 min to promote macrophage-bacteria interaction. Cells were then incubated for 30 min to allow for bacterial uptake. Afterward, apical media were replaced with an antibiotic cocktail (RPMI supplemented with 10% exosome-free FBS, 500 μg/mL gentamicin, and 500 U/mL penicillin–500 μg/mL streptomycin), and cells were incubated for 1 h to kill extracellular bacteria. Cells were then washed twice with PBS, with one duplicate well immediately lysed with 0.1% Triton X-100 to collect bacterial CFUs. CFUs were serially diluted and spotted on agar plates as a measurement of bacterial uptake. The remaining duplicate well was given fresh media and incubated for an additional 2 h before being lysed to collect CFUs as a measurement of bacterial survival. Results are calculated as Percent CFU Remaining [(Avg. CFU Survival / Avg. CFU Uptake) ∗ 100] or as Total CFU Counts [Log_10_(Avg. CFU)].

### Cell viability assay

LDH cytotoxicity assays were performed using the CytoTox 96 non-radioactive cytotoxicity assay (Promega) according to the manufacturer’s protocol.

### Cytokine analysis

Cytokine production by polarized 16HBE14o- epithelial cells and macrophages was evaluated via Bioplex using the Luminex Magpix multiplexing platform. Epithelial cell supernatants were analyzed using the Bio-Plex Pro Human Inflammation Panel-1 37-plex Assay (Bio-Rad), and macrophage supernatants were analyzed using the Bio-Plex Pro Human Cytokine 27-plex Assay (Bio-Rad), both according to the manufacturer’s protocol. Macrophage IL-6 production following EV treatment and *S. aureus* challenge was analyzed via Human IL-6 DuoSet ELISA (R&D) according to the manufacturer’s protocol.

### Flow cytometry

Immediately prior to poly(I:C) treatment, epithelial cells were stained as previously described with minor modifications (13). Briefly, cells were washed apically and basolaterally with PBS^+/+^ and incubated with 5 μM CellTrace Far Red (Invitrogen) in PBS^+/+^ for 30 min. Afterward, the dye was quenched by adding 1 mL of cell culture media and incubated for 10 min. Apical and basolateral compartments were then washed 2×, and the basolateral media were replaced with EV-free growth medium. Poly(I:C) treatment and EV isolation were performed as described above to yield fluorescent CEVs and AEVs. Macrophages were treated with EVs as previously described for 1 h before processing for flow cytometry analysis. For flow cytometry analysis, macrophages were lifted by placing cells on ice and shaking for 20 min in ice-cold PBS supplemented with 10 mM ethylenediaminetetraacetic acid (EDTA). Cells were stained with Live/Dead Zombie NIR (Biolegend), CD204 (Alexa-Fluor 700; Invitrogen), TLR2 (FITC; Invitrogen), CD36 (PerCP-eFluor 710; Invitrogen), CD206 (AF700; Invitrogen), Arginase 1 (eFluor 450; Invitrogen), and CD163 (PE-Cyanine 7; Invitrogen) in fluorescence-activated cell sorting (FACS) buffer (PBS^−/−^ supplemented with 2% FBS and 1 mM EDTA). Cells were fixed and permeabilized using Cytofix/CytoPerm (BD Biosciences) according to the manufacturer’s protocol. Cells were suspended in FACS buffer supplemented with 4% paraformaldehyde prior to assessment via Cytek Aurora. All analysis was performed using FlowJo analysis software.

### Label-free quantitative mass spectrometry

EVs isolated from a total of 1.2  ×  10^7^ 16HBE14o- cells were collected and lysed with a buffer containing 5% SDS and 50 mM triethylammonium bicarbonate that was supplemented with Roche Complete C protease and PhosSTOP phosphatase inhibitors. All EV samples were normalized to protein concentration, reduced, and alkylated, followed by digestion using S-Trap columns (ProtiFi) and trypsin (Promega) at a ratio of 1:10 (wt/wt). Peptides were suspended in 0.1% formic acid and resolved with liquid chromatography tandem mass spectrometry using a system composed of a nanoACQUITY HPLC (Waters) in line with a Q Exactive HF mass spectrometer (Thermo Fisher). Solvents A (0.1% formic acid in HPLC-grade water) and B (0.1% formic acid in 100% acetonitrile) were used as the mobile phases. Peptides were then eluted from an Acquity BEH C18 column, 1.7 µM particle size, 300 Å column (Waters) using a 48-min gradient at a flow rate of 0.9 µL/min (4% B for 8.1 min, 4%–7% B at 10  min, 7%–33% from 10 to 30 min, 33%–90% B from 30 to 33 min, 90% B for 3 min, 90%–4% B from 36 to 37 min, 4% B from 37.1 to 48 min to equilibrate the column). Data were collected in positive ionization mode, and the top 17 ions were subjected to high-energy collisional dissociation.

Peptide sequencing and identification were performed using PEAKSX (Bioinformatics Solutions, Inc.), a commercially available proteomics software that identifies peptides via a *de novo* peptide sequencing-assisted search engine database (54). Peptides in each sample were sequenced and identified using a decoy search at a 1% false discovery rate and the PEAKS peptide-spectrum matching score (−10lgP score) compared against the UniProt *Homo sapiens* (Human) database. For proteins identified in both conditions for all replicates, label-free quantification was performed by evaluating the weighted sum of peptide peak areas for each protein via the PEAKSQ add-on in the PEAKSX software. The proteomics data set has been deposited with the ProteomeXchange Consortium via the PRIDE partner repository with the data set identifier PXD041307.

To evaluate proteomic alterations between the two groups, we compared the average peak area in control vs antiviral EVs as analyzed by Log_2_(Fold Change) and -Log10(*P*-value) as determined by a two-tailed Student’s *t*-test. Proteins with Log_2_(Fold Change) and -Log10(*P*-value) ≥2 were considered significant. Significant proteins were analyzed for enrichment by KEGG pathways and for Gene Ontology categories via the Functional Annotation Clustering Tool in the Database for Annotation, Visualization and Integrated Discovery (DAVID Bioinformatics Resource 6.8). Enrichment analysis in DAVID used a modified Fisher’s exact test to determine whether proteins were enriched in the annotation categories.

### Mitochondrial stress test

Using a Seahorse XFe96 Bioanalyzer (Agilent), macrophages (8.0  ×  10^4^ per well) were plated on Seahorse culture plates in a medium consisting of minimal, unbuffered Dulbecco’s modified Eagle’s medium supplemented with 10  mmol L^−1^ of glucose, 1  mmol L^−1^ of pyruvate, and 2  mmol L^−1^ of glutamine. Basal OCRs were taken for 30  min. Cells were stimulated with 2  µmol L^−1^ of oligomycin, 0.5  µmol L^−1^ of FCCP, 10  mmol L^−1^ of 2-deoxyglucose, and 0.5  µmol L^−1^ of rotenone/antimycin A to obtain maximal respiratory and control values.

### Quantification and statistical analysis

Experiments were performed at least three times, as indicated in the figure legends. Means were compared using two-tailed paired *t*-tests when two data sets were compared, and for multiple comparisons, RM one-way analysis of variance with Geisser-Greenhouse correction. Data are presented as mean ± SD or median ± range, as indicated. GraphPad Prism was used for statistical analysis. *P* < 0.05 was considered significant.

## SUPPLEMENTAL MATERIAL

Figure S1 mbio.00863-23-s0001.pdf Poly(I:C) treatment of AECs induces robust anti-viral cytokine production.

Figure S2 mbio.00863-23-s0002.pdf Antibiotic protection assay CFU counts.

Figure S3 mbio.00863-23-s0003.pdf Gating strategy for flow cytometry analysis of EV-treated macrophages.

Figure S4 mbio.00863-23-s0004.pdf Characterization of macrophage polarization following EV treatment and *S. aureus* challenge.

Figure S5 mbio.00863-23-s0005.pdf Validation of TEPP-46 treatments.

Figure S6 mbio.00863-23-s0006.pdf Extracellular flux analysis of macrophage extracellular acidification rate (ECAR).

ASM does not own the copyrights to Supplemental Material that may be linked to, or accessed through, an article. The authors have granted ASM a non-exclusive, world-wide license to publish the Supplemental Material files. Please contact the corresponding author directly for reuse.
