# Supplementary material for: Antiviral epithelial-macrophage crosstalk permits secondary bacterial infections
Source: mBio. 2023 Sep 29;14(5):e00863-23. doi: 10.1128/mbio.00863-23 (PMC10653878; doi:10.1128/mbio.00863-23)
Supplement: Figure S1 — Poly(I:C) treatment of AECs induces robust anti-viral cytokine production. [file mbio.00863-23-s0001.pdf]

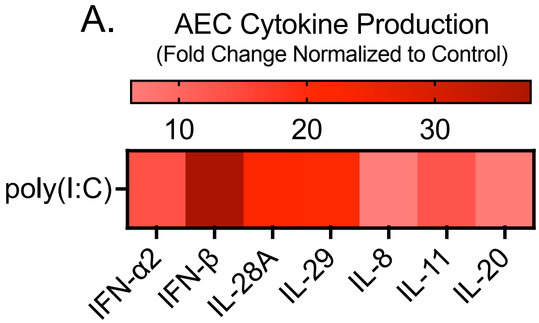

Supplemental Figure 1: Poly(I:C) treatment of AECs induces robust anti-viral cytokine production. (A) 37-plex cytokine array analysis of AEC supernatant following 18-hour poly(I:C) stimulation. Fold change calculated from detected concentration for each cytokine. All cytokines with a fold-change ≥ 5 displayed. Data displayed as mean; n ≥ 4.
