# Supplementary material for: Antiviral epithelial-macrophage crosstalk permits secondary bacterial infections
Source: mBio. 2023 Sep 29;14(5):e00863-23. doi: 10.1128/mbio.00863-23 (PMC10653878; doi:10.1128/mbio.00863-23)
Supplement: Figure S2 — Antibiotic protection assay CFU counts. [file mbio.00863-23-s0002.pdf]

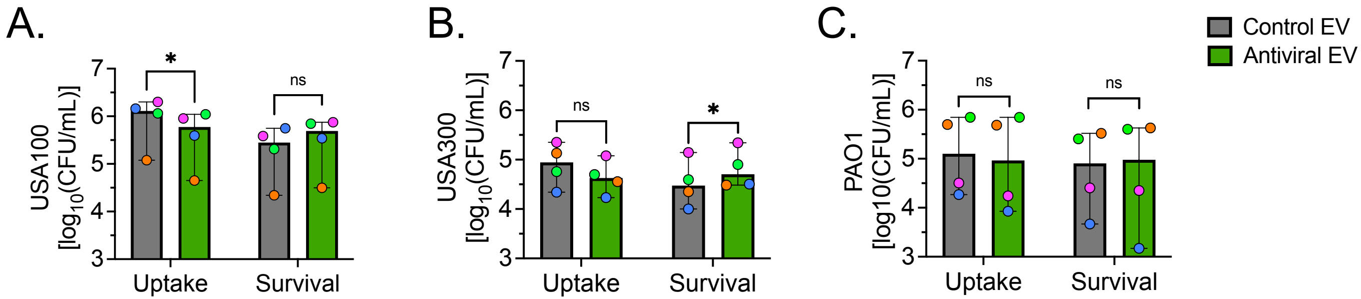

Supplemental Figure 2: Antibiotic protection assay CFU counts (Log_{10}) of (A) USA100, (B) USA300, and (C) PAO1. Grey bars: Control EVs; Green bars: Antiviral EVs. Analyzed via paired t-test, data displayed as median ± range, *P < .05. For all experiments, n ≥ 4 paired donors, each colored symbol denotes a donor.
