# Supplementary material for: Antiviral epithelial-macrophage crosstalk permits secondary bacterial infections
Source: mBio. 2023 Sep 29;14(5):e00863-23. doi: 10.1128/mbio.00863-23 (PMC10653878; doi:10.1128/mbio.00863-23)
Supplement: Figure S3 — Gating strategy for flow cytometry analysis of EV-treated macrophages. [file mbio.00863-23-s0003.pdf]

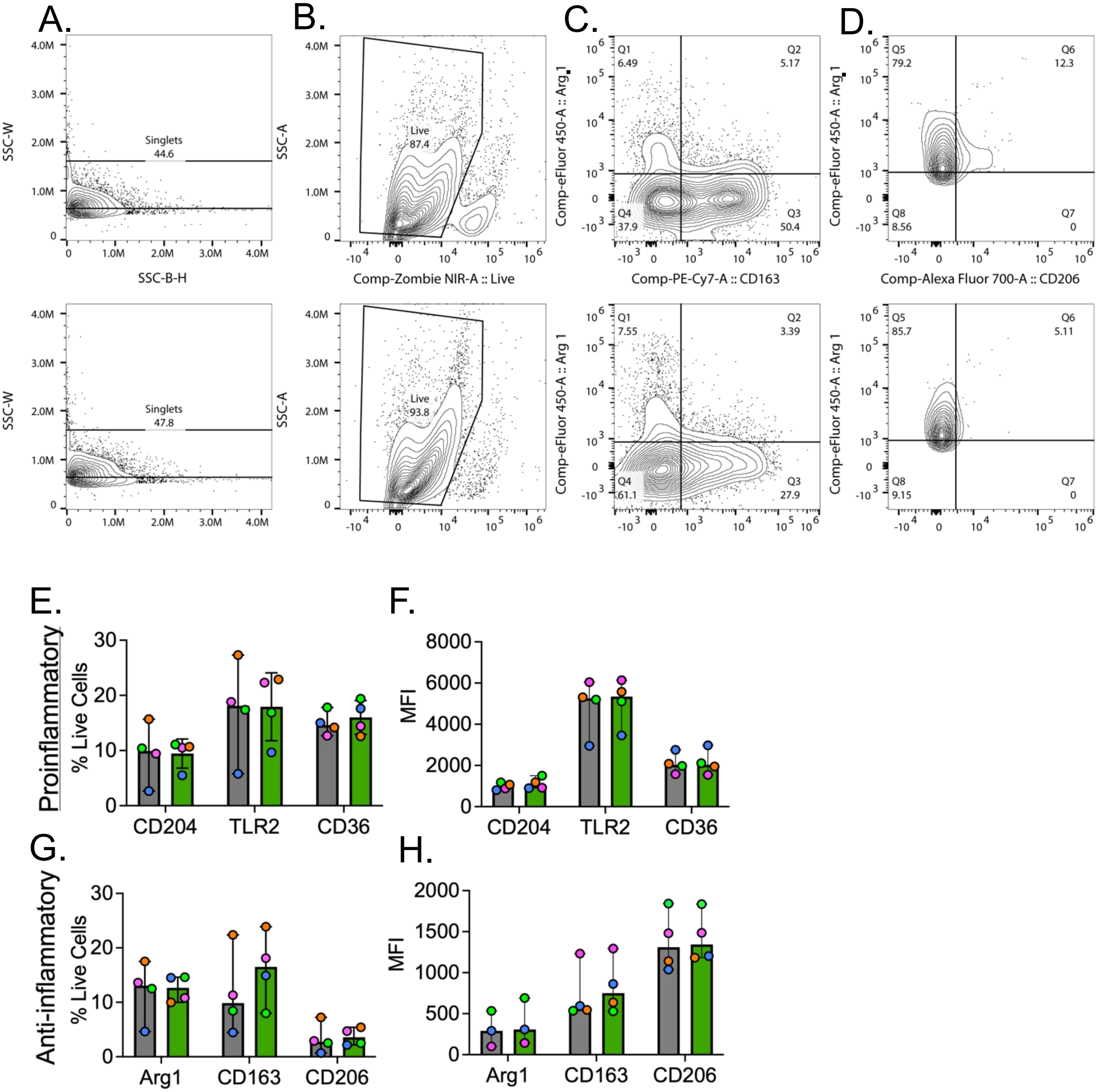

Supplemental Figure 3: Gating strategy for flow cytometry analysis of EV-treated macrophages. Representative flow plots for CEV and AEV treated macrophages. (A) Doublet cells were excluded from analysis. (B) Gating for Live cells, pre-gated on panel A. (C) Gating for Arg1 and CD163 pre-gated on panel B. (D) Gating for Arg1+CD163+ and CD206, pre-gated on panel C. Expression of single surface markers and relative MFI (geometric mean) following 18H EV treatment for proinflammatory (E-F) and anti-inflammatory (G-H) markers. Each color denotes a donor. Data displayed as median ± range.
