# Supplementary material for: Antiviral epithelial-macrophage crosstalk permits secondary bacterial infections
Source: mBio. 2023 Sep 29;14(5):e00863-23. doi: 10.1128/mbio.00863-23 (PMC10653878; doi:10.1128/mbio.00863-23)
Supplement: Figure S4 — Characterization of macrophage polarization following EV treatment and S. aureus challenge. [file mbio.00863-23-s0004.pdf]

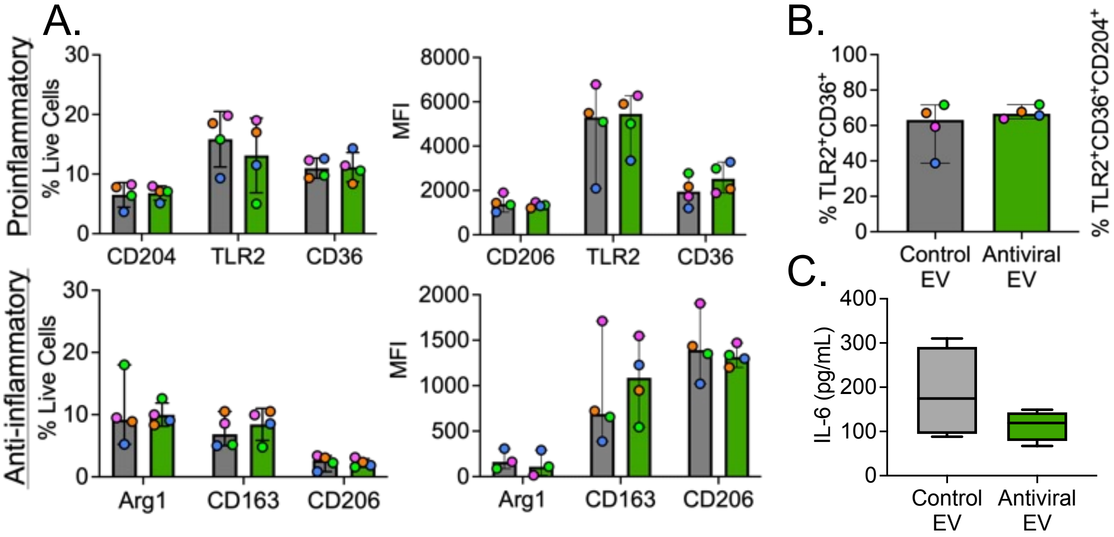

Supplemental Figure 4: Characterization of macrophage polarization following EV treatment and *S. aureus* challenge. Flow cytometry analysis of EV treated macrophages following S. aureus challenge showing (A) surface marker expression and relative MFI (geometric mean) (B) Percent of $TLR2^+CD36^+$ and $TLR2^+CD36^+CD204^+$ cells. Gated on frequency of live cells. Data displayed as bar graphs with median ± range (C) Macrophage supernatant was collected at the “Survival” timepoint of an antibiotic protection assay with USA100 and analyzed for IL-6 production via ELISA. Grey bars: Control EVs; Green bars: Antiviral EVs. Data displayed as a box plot with the black horizontal line representing the median, whiskers extend to minimum and maximum values. Analyzed via paired t-test, $^*P < .05$. For all experiments, $n \geq 4$ paired donors.
