# Supplementary material for: Antiviral epithelial-macrophage crosstalk permits secondary bacterial infections
Source: mBio. 2023 Sep 29;14(5):e00863-23. doi: 10.1128/mbio.00863-23 (PMC10653878; doi:10.1128/mbio.00863-23)
Supplement: Figure S5 — Validation of TEPP-46 treatments. [file mbio.00863-23-s0005.pdf]

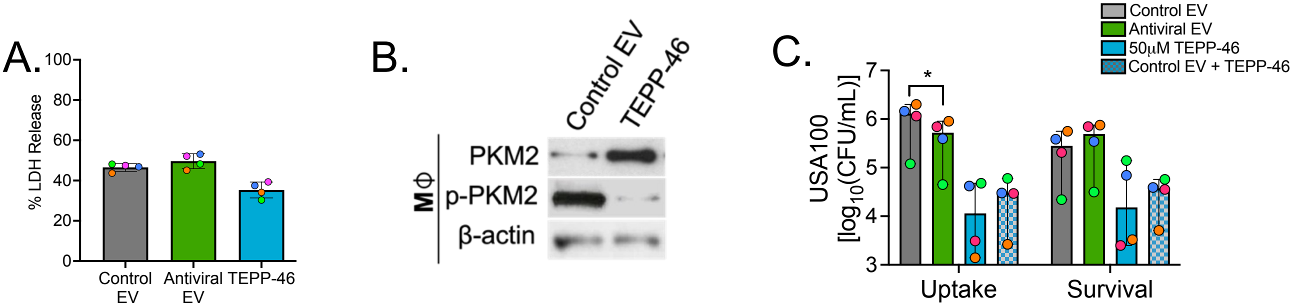

Supplemental Figure 5: (A) Macrophage viability after TEPP-46 treatment as assessed by LDH release. (B) Macrophage PKM2 expression following 24hr TEPP-46 treatment. (C) Antibiotic protection assay CFU counts (Log_{10}) of USA100 ± Control EVs, Antiviral EVs, or TEPP-46 treatment. Grey bars: Control EVs; Green bars: Antiviral EVs; Blue bars: TEPP-46; Blue checkered bars: Control EVs +TEPP-46 treatment. Each colored symbol denotes a donor. Multiple comparisons analyzed by RM one-way ANOVA with Geisser-Greenhouse correction, data displayed as median ± range. For all experiments, n ≥ 4 paired donors.
