# Supplementary material for: Antiviral epithelial-macrophage crosstalk permits secondary bacterial infections
Source: mBio. 2023 Sep 29;14(5):e00863-23. doi: 10.1128/mbio.00863-23 (PMC10653878; doi:10.1128/mbio.00863-23)
Supplement: Figure S6 — Extracellular flux analysis of macrophage extracellular acidification rate (ECAR). [file mbio.00863-23-s0006.pdf]

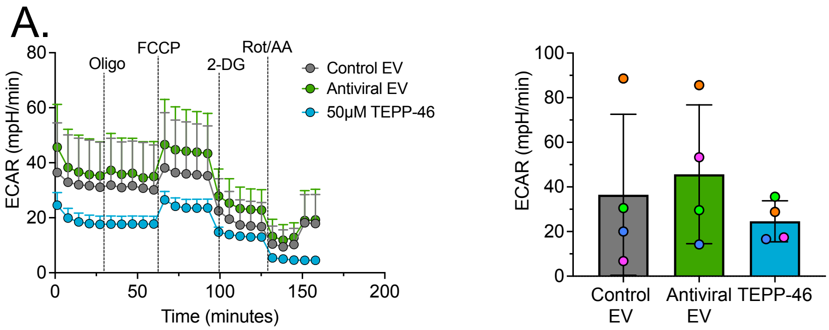

Supplemental Figure 6: Extracellular flux analysis of macrophage extracellular acidification rate (ECAR) after pretreatment with EVs or TEPP-46. (A) Kinetic graph and quantification of macrophage ECAR. Each colored symbol denotes a donor. Analyzed by RM one-way ANOVA with Geisser-Greenhouse correction. Data displayed as median ± range. For all experiments, n ≥ 4 paired donors.
